# Timing of Full Enteral Feeding and Longitudinal Gut Microbiome Development in Infants Admitted to the Neonatal Intensive Care Unit

**DOI:** 10.3390/nu18193262

**Published:** 2026-10-02

**Authors:** Jeongmin Shin, Hyun Ho Kim, Sae Yun Kim, Hyun Mi Kang, Young-Ah Youn

**Affiliations:** Department of Pediatrics, Seoul St. Mary’s Hospital, College of Medicine, The Catholic University of Korea, 222 Banpo-daero, Seocho-gu, Seoul 06591, Republic of Korea; emmyshin@catholic.ac.kr (J.S.); gushkrs@gmail.com (H.H.K.); pedhmk@catholic.ac.kr (H.M.K.); lea732@hanmail.net (Y.-A.Y.)

**Keywords:** gut microbiome, preterm infant, full enteral feeding, 16S rRNA

## Abstract

Background/Objectives: Delayed achievement of full enteral feeding (FEF) is common in neonatal intensive care unit (NICU) infants but is closely linked to gestational age (GA). We examined whether time to FEF is associated with gut microbiome development from birth to 6 months of corrected age (CA) beyond GA. Methods: We profiled 694 stool samples from 328 NICU infants, collected at first stool, discharge, and 4- and 6-month CA, by *16S rRNA* gene sequencing. Infants were classified as Early FEF (<14 days, n = 293) or Delayed FEF (≥14 days, n = 35), and time to FEF was also analyzed continuously. Linear mixed models and PERMANOVA were adjusted for GA. Results: Composition at first stool did not differ between groups. At discharge, the Delayed group had higher Proteobacteria, lower Firmicutes, lower *Streptococcus* (*q* = 0.028), and a different community structure in unadjusted analyses, but none of these differences remained after GA adjustment. No phylum showed a GA-adjusted trajectory difference, and alpha diversity did not differ. In an exploratory continuous analysis, longer time to FEF was associated with higher Bacteroidetes at discharge and 4-month CA. Conclusions: Most microbiome differences associated with delayed FEF were not independent of GA. Time to FEF may reflect maturity and illness severity rather than act as an independent determinant of the gut microbiome.

## 1. Introduction

The neonatal gut microbiome is established rapidly after birth. Its developmental trajectory during early life is critical for immune maturation, metabolic programming, and intestinal mucosal barrier function [1,2]. Infants admitted to the neonatal intensive care unit (NICU), particularly those born preterm, are exposed to multiple disruptive factors, including cesarean delivery, broad-spectrum antibiotics, prolonged parenteral nutrition, and delayed enteral feeding, all of which impair normal microbial colonization [3,4,5].

Gestational age (GA) is among the strongest determinants of gut microbiome assembly. Lower GA is associated with Proteobacteria predominance and delayed *Bifidobacterium* colonization [6,7]. This dysbiotic pattern is closely linked to necrotizing enterocolitis (NEC) and late-onset sepsis [8,9]. Lower GA also independently delays achievement of full enteral feeding (FEF). However, because lower GA is strongly associated with delayed achievement of full enteral feeding, it remains unclear whether altered gut microbiome development in NICU infants primarily reflects biological immaturity, delayed enteral nutritional exposure, or both.

FEF achievement is a key clinical milestone. A large trial found that faster feed advancement did not reduce late-onset sepsis [10], but in observational data, later FEF has been associated with greater morbidity and a higher risk of late-onset sepsis [11]. Enteral nutrition provides luminal substrates required for epithelial growth, mucosal immune development, and obligate anaerobic colonization [5,12]. Thus, earlier establishment of enteral nutrition may facilitate the transition from an early facultative anaerobe-dominated community toward a more mature anaerobe-rich microbial ecosystem [13]. Delayed feeding may therefore influence microbial succession not only by limiting nutrient delivery, but also by delaying luminal substrate availability and the ecological conditions necessary for anaerobic colonization.

Although several longitudinal studies of the preterm gut microbiome exist [6,14,15,16], few have evaluated microbiome development at multiple clinically relevant timepoints from birth through corrected infancy while simultaneously considering enteral feeding progression. Time to FEF is routinely recorded in NICU care and reflects both maturity and the clinical course, but whether it carries information about microbiome development beyond gestational age, and whether any association extends beyond discharge, remains unclear. This study aimed to characterize longitudinal gut microbiome trajectories from birth to 6 months corrected age in NICU infants and to determine whether the timing of FEF achievement is associated with microbial maturation across four timepoints, beyond the effects of gestational age.

## 2. Materials and Methods

### 2.1. Study Design and Population

We conducted a retrospective analysis of prospectively collected data from infants admitted to the NICU of a tertiary referral university hospital in Seoul, Korea (Seoul St. Mary’s Hospital, The Catholic University of Korea). The parent cohort enrolled 360 neonates admitted to the NICU between April 2022 and January 2024, and was reported previously [17]. That report described longitudinal changes in gut microbiome composition and diversity across gestational age groups from birth to 6 months of corrected age. The present analysis includes 694 of the 709 stool samples in that report that were obtained from infants with available FEF data, and it examines a different question, namely whether the timing of FEF is associated with microbiome development beyond gestational age. The inclusion criteria were: (1) admission to the NICU immediately after birth, regardless of GA; (2) written informed consent from legal guardians; and (3) availability of stool samples for microbiome analysis at one or more timepoints. Exclusion criteria included major congenital anomalies, chromosomal abnormalities, and inborn metabolic disorders. Full-term infants admitted solely for gastrointestinal problems were also excluded. Clinical data were obtained from the electronic medical record system. The study protocol was approved by the Human Research Ethics Board of Seoul St. Mary’s Hospital (IRB No. KC22RISI0240 and KC22TNSI0297). Informed consent was obtained from the parents of all enrolled infants.

### 2.2. Definitions

FEF was defined as tolerance of a target milk volume of ≥100 mL/kg/day. Infants who achieved FEF within 14 hospital days were classified as Early FEF, and those who required 14 days or more were classified as Delayed FEF. The 14-day threshold was based on the 90th percentile of the FEF distribution in this cohort and on a large multicenter cohort of extremely preterm infants, in which the median time to full enteral feeding was 14 days in recent years and each additional week of delay was associated with a higher risk of late-onset sepsis [11]. Because this threshold was partly derived from the distribution in this cohort, time to FEF was also analyzed as a continuous variable (Section 2.6).

Gestational age was determined by the last menstrual period or first-trimester ultrasound. Antenatal corticosteroids referred to the administration of one or more doses to the mother before delivery for fetal lung maturity. Premature rupture of membranes (PPROM) was defined as rupture lasting more than 18 h before delivery. Histological chorioamnionitis (HCAM) was diagnosed by acute inflammatory changes in placenta-related tissue [18]. Small for gestational age (SGA) was defined as birth weight below the 10th percentile for GA and sex [19]. Respiratory distress syndrome (RDS) was diagnosed by clinical and radiographic findings. Bronchopulmonary dysplasia (BPD) was classified according to the National Institute of Child Health consensus on BPD severity [20]. Sepsis was defined as a positive blood culture. Necrotizing enterocolitis (NEC) was diagnosed at modified Bell stage II or higher [21]. Feeding intolerance (FI) was defined as persistent gastric aspirates exceeding 50% of the feed volume, with or without increased abdominal girth, in the absence of sepsis or radiographic evidence of NEC; the condition had to last at least 48 h and occur more than three times per day, preventing advancement of feeding beyond 10 to 20 mL/kg/day [22]. Intraventricular hemorrhage (IVH) and periventricular leukomalacia (PVL) were diagnosed by cranial ultrasound. Retinopathy of prematurity (ROP) was defined as ROP requiring treatment, and untreated ROP was not counted [23,24]. Corrected age (CA) was calculated by subtracting the number of weeks born before 40 weeks of gestation from the chronological age.

### 2.3. Data Collection

Maternal and perinatal variables collected included maternal age, obstetric complications, PPROM, mode of delivery, and antenatal antibiotic use. Neonatal variables included GA, birth weight, and the following major morbidities: RDS, BPD, NEC or FI, IVH, PVL, and culture-proven sepsis. Feeding-related data collected included type of feeding (breast milk vs. formula), time to achievement of FEF, and duration of total parenteral nutrition (TPN). TPN duration was calculated as the total number of days during which intravenous amino acids or lipid emulsions were administered. Breast milk exposure was defined as breast milk accounting for more than 50% of the total enteral intake during admission. Neonatal antibiotic exposure was recorded as the number of days of administration of each agent during admission, and antibiotic days were calculated as the sum across agents. The dates of antibiotic administration were not available. Probiotics (BioGaia, *Limosilactobacillus reuteri* DSM 17938) were given as one drop once daily to all infants born before 32 weeks of gestation or weighing less than 1500 g, and to other infants at parental request. Probiotics were started with enteral feeding and continued until discharge. Data on feeding and probiotic use after discharge were not available.

### 2.4. Stool Sample Collection and Timepoints

Stool samples were collected at four timepoints: (1) first stool (meconium), collected within 72 h of birth; (2) last stool before discharge from the NICU; (3) 4-month CA; and (4) 6-month CA. Samples collected at the NICU were obtained by nursing staff as part of routine care. Approximately 1–3 mL of stool was collected using sterile technique and immediately frozen at −20 °C. Within 48 h, samples were transferred to a −80 °C deep freezer for storage until processing. Samples at 4- and 6-month CA were collected at home by parents using a stool collection kit containing transport medium (REST NBgene-GUT, Noblebiosciences, Hwaseong, Republic of Korea). These samples were kept at room temperature, delivered to the laboratory within 14 days of collection, and stored at −80 °C until DNA extraction. Postmenstrual age at the discharge sample was calculated as GA at birth plus postnatal age at sample collection.

### 2.5. 16S rRNA Gene Sequencing and Bioinformatics

Microbial DNA was extracted with a TIANLONG nucleic acid extraction kit for stool DNA/RNA (TianLong Science and Technology, Xi’an, China), and DNA concentration and purity were measured with a NanoDrop spectrophotometer (Thermo Fisher Scientific, Wilmington, DE, USA). The V3–V4 region of the bacterial 16S rRNA gene was amplified by polymerase chain reaction (PCR) with primers 341F (5′-CCTACGGGNGGCWGCAG-3′) and 805R (5′-GACTACHVGGGTATCTAATCC-3′) carrying Illumina overhang adapters (25 cycles), followed by dual-index PCR (8 cycles) using the PCRBIO VeriFi Mix (PCR Biosystems^®^, London, UK) and the Nextera^®^ Index Kit V2 Set A (Illumina^®^, San Diego, CA, USA). Amplicons were purified with the MagListo PCR/Gel Purification Kit (Bioneer, Daejeon, Republic of Korea) and quantified with Qsep100 (Bioptic, Changzhou, China) and a Qubit fluorometer ((Invitrogen, Thermo Fisher Scientific, Waltham, MA, USA)). Sequencing was performed on an Illumina MiSeq platform with the MiSeq Reagent Kit v3 (600 cycles, 2 × 300 bp paired-end reads), with PhiX as an internal control. Raw sequence data were processed using the Quantitative Insights Into Microbial Ecology 2 (QIIME2; version 2020.6) pipeline. Reads shorter than 200 bp were removed. Reads were demultiplexed and denoised with DADA2, with trimming at 15 bp and truncation at 260 bp, and chimeric reads were removed. Environmental contaminants were filtered with the R package decontam (version 1.8.0). Of 812 samples collected in the parent cohort, 103 were excluded because of poor preservation or inadequate sequencing quality [17]. Taxonomic profiles were generated with the 16S Metagenomics App (Illumina). Microbial composition was analyzed at the phylum and genus levels. Laboratory processing and bioinformatic analyses were performed without knowledge of the FEF group.

### 2.6. Statistical Analysis

Statistical analyses were performed using Python (v3.11, Python Software Foundation). Microbiome relative abundances were non-normally distributed, so cross-sectional comparisons of relative abundance used non-parametric tests. Continuous variables were compared with the Mann–Whitney U test, and categorical variables with the chi-square or Fisher’s exact test. Continuous clinical variables are presented as median (interquartile range, IQR). The correlation between GA and time to FEF was assessed with Spearman’s rank correlation. For phylum- and genus-level relative abundance comparisons, the Mann–Whitney U test was applied at each timepoint. Phylum-level cross-sectional comparisons and alpha diversity comparisons were not corrected for multiple testing and are reported as nominal *p* values. At the genus level, *p*-values were corrected using the Benjamini–Hochberg false discovery rate (FDR) across 19 genera with mean relative abundance ≥ 1% at four timepoints (76 tests). Significance at the genus level was set at *q* < 0.05. This genus-level screen was based on relative abundances and was not adjusted for GA. The genus that remained significant after correction was therefore re-examined with a compositional model adjusted for GA in the sensitivity analysis at discharge. For longitudinal trajectory analysis, phylum-level relative abundances were centered log-ratio (CLR)-transformed [25], with zero values replaced by a pseudocount of 0.0001 in all CLR analyses, and analyzed with linear mixed models fitted by restricted maximum likelihood (REML), specifying fixed effects for FEF group, timepoint, their interaction, GA, and GA × timepoint, and random intercepts per subject. The GA × timepoint terms allowed the association between GA and each phylum to differ by timepoint. A model with a single GA term showed associations between FEF timing and composition at first stool, which was collected before enteral feeding was established. The GA × timepoint terms were therefore added to reduce residual confounding by GA, and the results of the single-term model are summarized in Table S3. The FEF × timepoint interaction coefficient quantified the between-group trajectory difference. Joint Wald tests of the interaction terms were corrected across the five phyla, and timepoint-specific contrasts were corrected across the 20 phylum and timepoint combinations, using the Benjamini–Hochberg method. As a sensitivity analysis, time to FEF was analyzed as a continuous exposure on the log2 scale in the same model. Alpha diversity (Shannon index, number of species identified) was compared with the Mann–Whitney U test. Beta diversity was assessed by Bray–Curtis dissimilarity with principal coordinates analysis (PCoA) and tested by PERMANOVA, both without adjustment and with GA as a covariate using Freedman–Lane permutation of residuals (9999 permutations), with PERMDISP for within-group dispersion. Significance was set at *p* < 0.05. The linear mixed models used all available samples and provide valid estimates when missingness depends only on variables included in the model. GA and FEF groups, which were associated with follow-up, were included in all models. A second sensitivity analysis was performed at discharge. CLR-transformed *Streptococcus* abundance, computed across the 19 genera and the sum of the remaining genera, and the CLR-transformed abundance of the five phyla were compared by linear regression with heteroscedasticity-consistent (HC3) standard errors. Beta diversity was tested by PERMANOVA with Freedman–Lane permutation of residuals from the covariate-only model (9999 permutations). Models were fitted in the full cohort with adjustment for GA, and in infants born before 34 weeks of gestation with adjustment for GA and breast milk exposure.

Covariate selection was based on the assumed causal relationships between FEF timing, clinical exposures, and the gut microbiome. GA was considered the principal confounder because it precedes FEF achievement and independently shapes microbial colonization. Duration of parenteral nutrition, length of stay, and postmenstrual age at the discharge sample were regarded as consequences of delayed FEF and were not adjusted for. Major neonatal morbidities were considered both causes and consequences of delayed feeding, and NEC and feeding intolerance overlap with the definition of delayed FEF. Adjustment for these variables could remove part of the association of interest, so they were not included as covariates [26]. Antibiotic exposure may act both as a confounder and as a consequence of delayed FEF. It was strongly correlated with time to FEF, and without administration dates, the exposure preceding each sample could not be defined. Antibiotic exposure was therefore not included as a covariate and is reported descriptively. Probiotic and breast milk exposure was cumulative over the admission and corresponded in time only to the discharge sample, so these exposures were addressed in the sensitivity analysis at discharge.

## 3. Results

### 3.1. Study Population and Demographics

**Figure 1:**
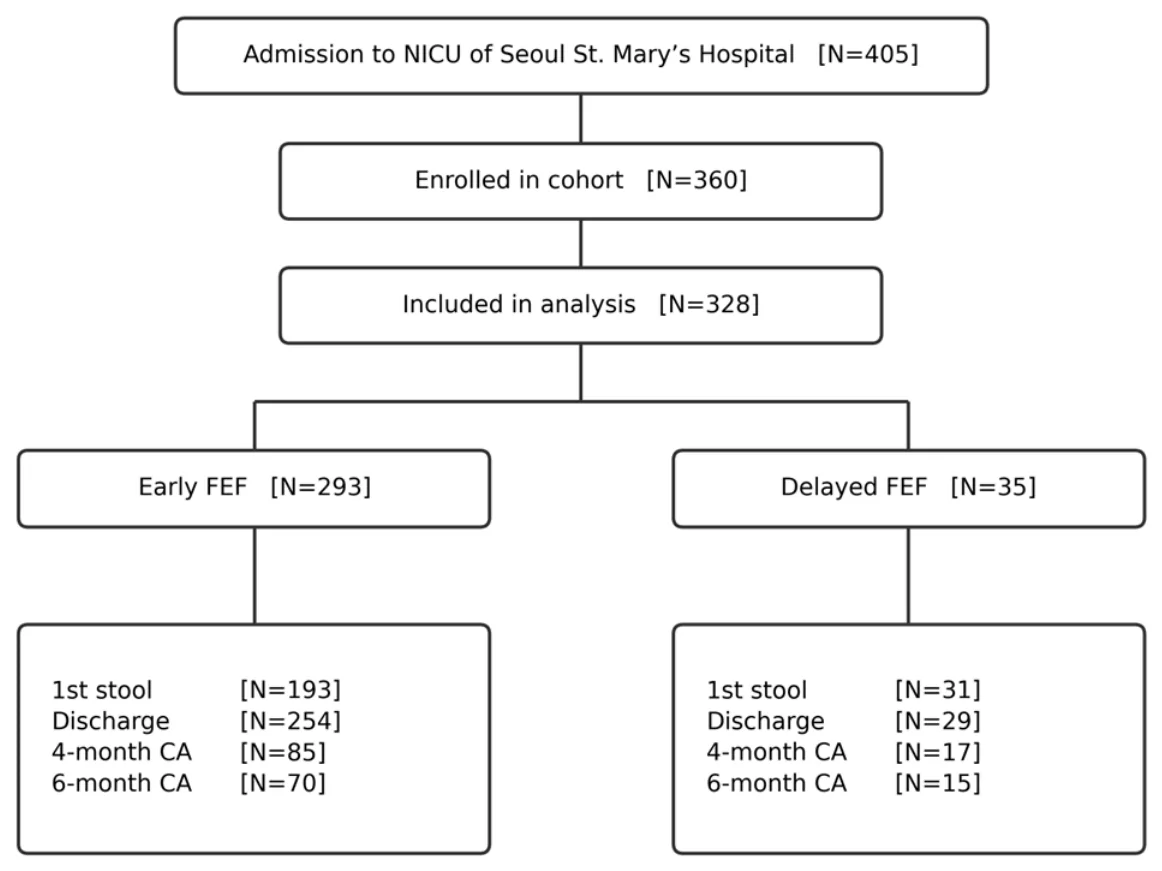
Study population flow diagram. Of the 360 neonates enrolled in the parent cohort, 328 with full enteral feeding (FEF) data and at least one stool sample were included and classified as Early FEF (<14 hospital days, n = 293) or Delayed FEF (≥14 hospital days, n = 35). The number of stool samples analyzed at each timepoint is shown. Abbreviations: FEF, full enteral feeding; NICU, neonatal intensive care unit.

Among the 360 enrolled neonates, 328 infants with complete clinical information, including FEF achievement data, and at least one stool sample for microbiome analysis were included in the final analysis, contributing a total of 694 stool samples. Of these, 293 (89.3%) achieved FEF within 14 hospital days (Early FEF group), and 35 (10.7%) required 14 or more days (Delayed FEF group). The study population is described in Figure 1. Between-group comparisons of demographic and clinical characteristics are presented in Table 1. The Delayed group had lower GA (median 27.9 [IQR 26.2–30.0] vs. 35.3 [33.9–37.4] weeks, *p* < 0.001) and birth weight (1005 [762.5–1339] vs. 2370 [2011–2910] g, *p* < 0.001). Hospital stay (109 [68.5–138.5] vs. 15 [10–25] days), mechanical ventilation duration (11 [2–30.5] vs. 0 [0–2] days), and TPN duration (36 [25.5–56.5] vs. 3 [2–5] days) were all longer (all *p* < 0.001). Antenatal steroid use (97.1% vs. 53.9%, *p* < 0.001) and histological chorioamnionitis (17.1% vs. 2.0%, *p* < 0.001) were also more frequent. PPROM was more common in the Delayed group (28.6% vs. 11.6%, *p* = 0.005). Major morbidities were more prevalent in the Delayed group: RDS (82.9% vs. 25.6%), symptomatic PDA (42.9% vs. 0%), BPD (37.1% vs. 1.0%), NEC or feeding intolerance (28.6% vs. 7.8%), sepsis (25.7% vs. 1.0%), any IVH (97.1% vs. 64.2%), IVH grade ≥ 2 (42.9% vs. 0.7%), and PVL (80.0% vs. 22.5%) (all *p* < 0.001). Breast milk receipt > 50% was more frequent in the Delayed group (37.1% vs. 2.4%, *p* < 0.001). All infants in the Delayed group and 66.2% of infants in the Early group received probiotics. Antibiotics were given to nearly all infants in both groups (100% vs. 98.0%, *p* = 1.000), but antibiotic days were longer in the Delayed group (45 [30.5–95.5] vs. 8 [7–11] days, *p* < 0.001). Sex and cesarean delivery rates did not differ. At the discharge sample (n = 275 with available dates), postnatal age was 95 [65–128] vs. 14 [8–22] days and postmenstrual age was 41.9 [38.6–45.7] vs. 37.8 [36.7–39.0] weeks in the Delayed and Early groups (both *p* < 0.001). Time to FEF was inversely correlated with GA (Spearman ρ = −0.62, *p* < 0.001). Of the 328 infants, 113 (34.5%) contributed at least one sample at 4-month or 6-month CA. Compared with infants without a post-discharge sample, these infants had a lower GA (median 34.4 vs. 35.3 weeks, *p* = 0.015) and were more often in the Delayed group (16.8% vs. 7.4%, *p* = 0.015). Sex and mode of delivery did not differ.

### 3.2. Phylum-Level Gut Microbiome Composition

**Figure 2:**
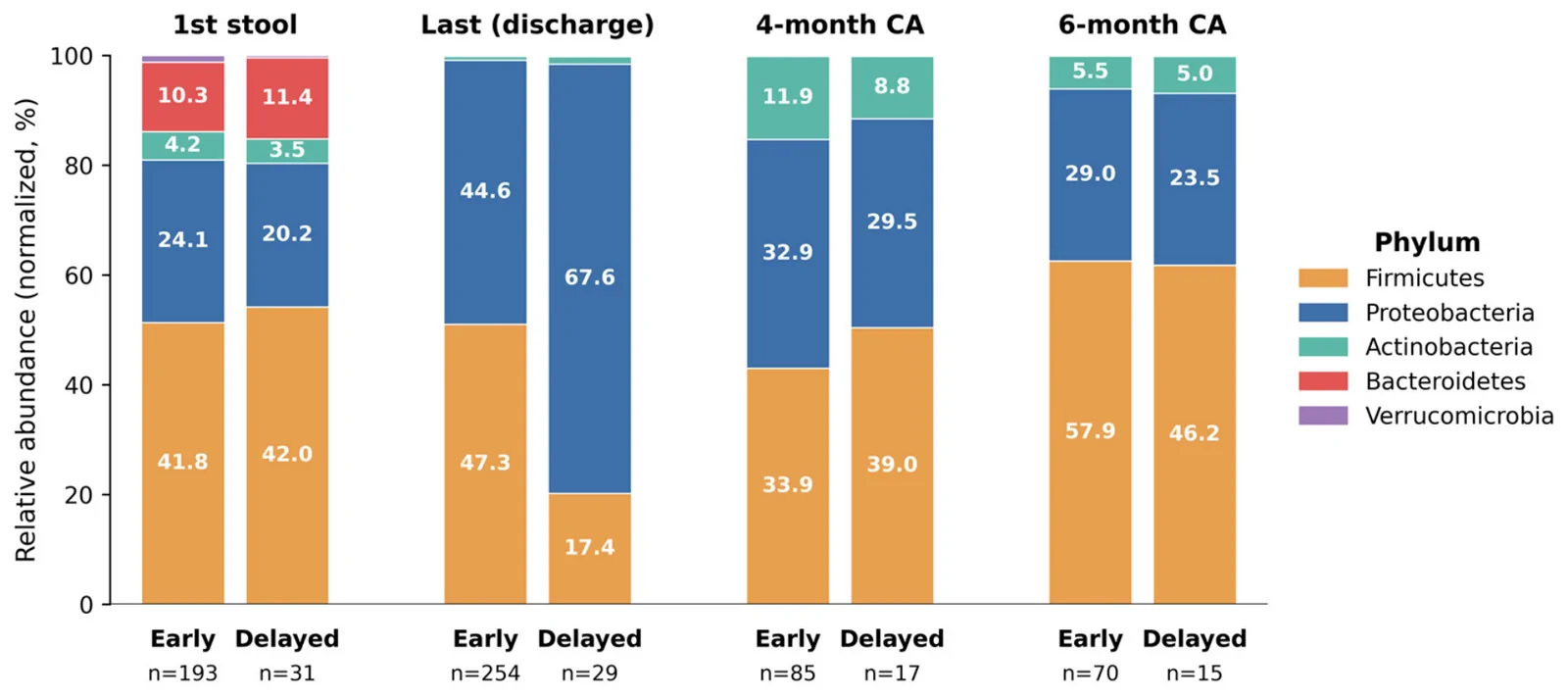
Phylum-level gut microbiome composition in the Early FEF and Delayed FEF groups across four timepoints. Each stacked bar shows the median relative abundance of the five phyla listed in the legend, normalized so that the five phyla sum to 100%. The four timepoints are first stool, Last (discharge), 4-month CA, and 6-month CA. The number within each segment is the median relative abundance (%) of that phylum before normalization. The number of infants contributing samples at each timepoint is shown below each bar. Abbreviations: CA, corrected age; FEF, full enteral feeding.

Phylum-level relative abundances at each timepoint are presented in Figure 2 and Table S1. At first stool, composition was comparable between groups across all five phyla (all *p* > 0.05). By discharge, the Delayed group had higher Proteobacteria (59.5 ± 34.3% vs. 43.8 ± 37.9%, *p* = 0.035) and lower Firmicutes (32.0 ± 31.0% vs. 50.2 ± 36.2%, *p* = 0.012) in unadjusted comparisons without correction for multiple testing. At 4-month CA, Actinobacteria was numerically higher in the Early group (23.8 ± 25.2% vs. 13.6 ± 14.8%, *p* = 0.269), while Bacteroidetes (*p* = 0.578) and Verrucomicrobia (*p* = 0.615) were numerically higher in the Delayed group without reaching significance. By 6-month CA, no significant differences were observed for any phylum (all *p* > 0.05), although sample sizes after discharge were smaller.

### 3.3. Genus-Level Gut Microbiome Composition

**Figure 3:**
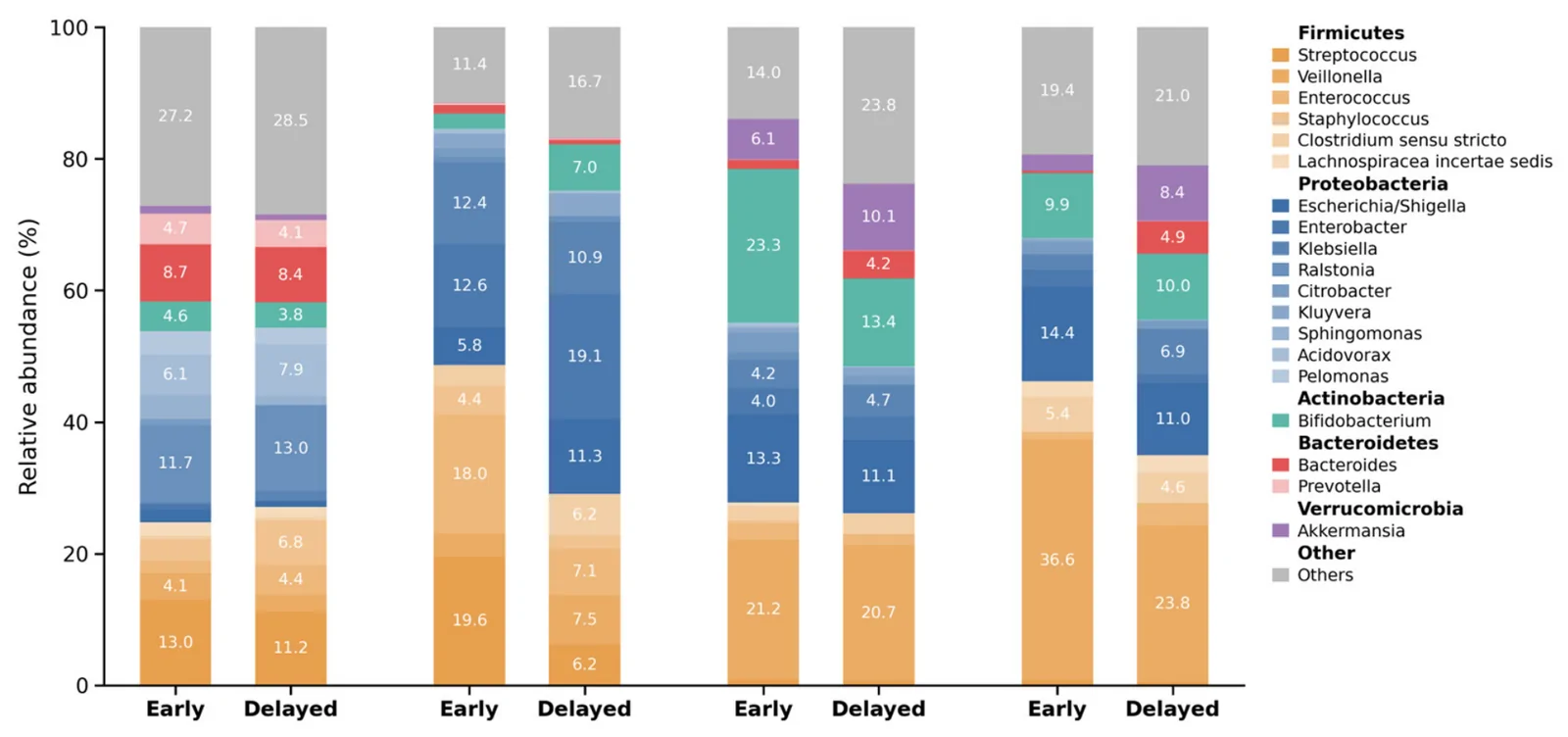
Genus-level gut microbiome composition in the Early FEF and Delayed FEF groups across four timepoints. Each stacked bar shows the mean relative abundance (%) of the genera listed in the legend, grouped and colored by phylum. The number within a segment is the mean relative abundance for that genus. The remaining abundance up to 100% corresponds to other genera grouped as Others. Abbreviations: CA, corrected age; FEF, full enteral feeding.

Genus-level compositions at each timepoint are shown in Figure 3 and Table S2. At first stool, both groups showed similar profiles dominated by *Streptococcus*, *Ralstonia*, and *Bacteroides*. By discharge, the two groups differed in their dominant taxa. The Early group was dominated by *Streptococcus* (19.6%) and *Enterococcus* (18.0%), whereas the Delayed group was dominated by *Enterobacter* (19.1%) and *Escherichia/Shigella* (11.3%). At 4-month CA, *Bifidobacterium* was the most abundant genus in the Early group (23.3% vs. 13.4%), consistent with the higher Actinobacteria abundance at the phylum level, while *Akkermansia* (6.1% vs. 10.1%) and *Bacteroides* (1.4% vs. 4.2%) were more abundant in the Delayed group. By 6-month CA, both groups shifted to a *Veillonella*-dominated profile, and *Streptococcus* had declined to below 1% in both groups.

Nineteen genera with a mean relative abundance of at least 1% across the cohort were compared between groups at each of the four timepoints, giving 76 tests. Six comparisons reached nominal significance. After Benjamini–Hochberg correction, only *Streptococcus* at discharge remained significant (Early 19.6% vs. Delayed 6.2%, *p* < 0.001, *q* = 0.028). This difference was attenuated in the GA-adjusted compositional analysis (Section 3.7). *Staphylococcus* at discharge (*p* = 0.017, *q* = 0.280), *Veillonella* at 6-month CA (*p* = 0.018, *q* = 0.280), and *Bifidobacterium* at discharge, which was higher in the Delayed group (2.3% vs. 7.0%, *p* = 0.030, *q* = 0.379), did not survive correction. *Enterobacter* at first stool (*p* = 0.013, *q* = 0.280) and *Sphingomonas* at 4-month CA (*p* = 0.005, *q* = 0.178) also reached nominal significance but did not survive correction.

### 3.4. Longitudinal Trajectory of Phylum-Level Composition

**Figure 4:**
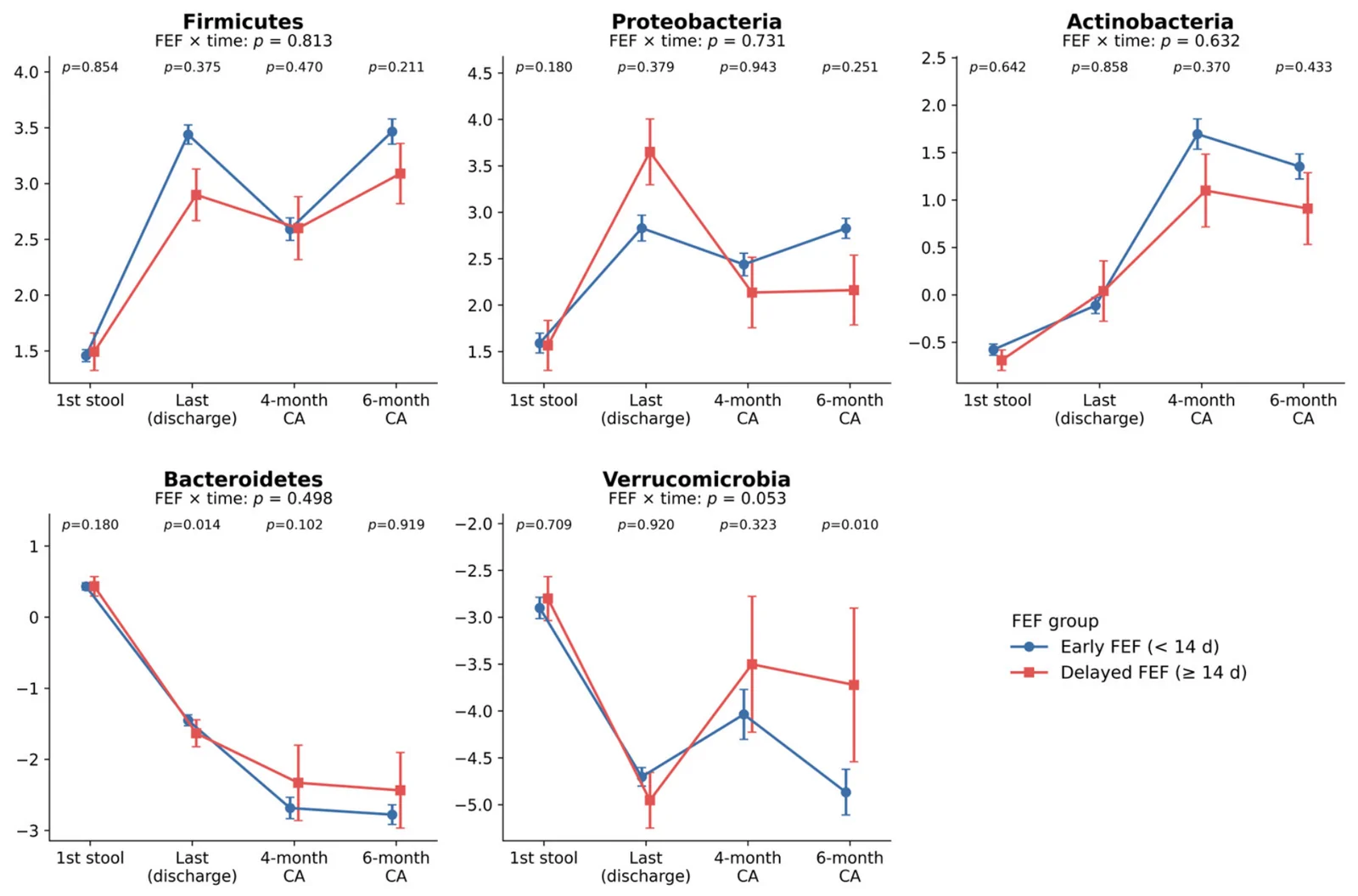
GA-adjusted longitudinal trajectories of phylum-level gut microbiome composition in the Early FEF and Delayed FEF groups. Each panel shows the centered log-ratio (CLR)-transformed relative abundance of one phylum across the four timepoints (first stool, Last [discharge], 4-month CA, and 6-month CA). Points are unadjusted group means, and error bars represent the standard error of the mean. The *p* value above each timepoint is the gestational age-adjusted between-group contrast from a random-intercept linear mixed model adjusted for GA and GA × timepoint, and the FEF × time *p* value in each panel header is the joint test of the group-by-timepoint interaction terms in the same model. All *p* values are nominal, and none remained significant after Benjamini–Hochberg correction. Abbreviations: CA, corrected age; CLR, centered log-ratio; FEF, full enteral feeding.

Phylum-level relative abundances were centered log-ratio-transformed and analyzed with a random-intercept linear mixed model adjusted for GA and GA × timepoint. Group trajectories across the four timepoints are shown in Figure 4 and Table S3. Both groups followed the same overall developmental pattern. Firmicutes and Proteobacteria rose from first stool to discharge, Actinobacteria rose after discharge and peaked at 4-month CA, and Bacteroidetes and Verrucomicrobia declined from first stool to discharge before partially recovering.

The FEF group by time interaction, which tests whether the shape of the trajectory differed between groups, was not significant for any phylum after GA adjustment (Firmicutes *p* = 0.813, Proteobacteria *p* = 0.731, Actinobacteria *p* = 0.632, Bacteroidetes *p* = 0.498, and Verrucomicrobia *p* = 0.053), and none remained significant after Benjamini–Hochberg correction across the five phyla (all *q* ≥ 0.264).

GA-adjusted between-group contrasts at individual timepoints reached nominal significance in two instances. Bacteroidetes was higher in the Delayed group at discharge (*p* = 0.014), and Verrucomicrobia was higher in the Delayed group at 6-month CA (*p* = 0.010). No contrast at first stool differed between groups (all *p* ≥ 0.180). None of the 20 contrasts remained significant after Benjamini–Hochberg correction (minimum *q* = 0.140). In the model with a single GA term, contrasts at first stool were nominally different for Proteobacteria (*p* = 0.039) and Bacteroidetes (*p* = 0.021) (Table S3).

Taken together, the phylum-level trajectories did not diverge significantly between the Early group and the Delayed group once GA was taken into account. The cross-sectional differences observed at discharge in the unadjusted analysis were attenuated after GA adjustment, which is consistent with the strong inverse correlation between GA and time to FEF in this cohort (ρ = −0.62).

When time to FEF was analyzed as a continuous variable, the FEF × time interaction was not significant for any phylum after correction (all *q* ≥ 0.387; Table S3). Longer time to FEF was associated with higher Bacteroidetes at discharge (β per doubling of days 0.28, 95% CI 0.09 to 0.48, *q* = 0.045) and at 4-month CA (β 0.74, 95% CI 0.35 to 1.13, *q* = 0.004). The estimate at 6-month CA was similar in size (β 0.54, 95% CI 0.13 to 0.94, *q* = 0.065). At first stool the estimate was in the same direction but smaller (β 0.20, 95% CI −0.03 to 0.44, *p* = 0.093). No other phylum was associated with time to FEF at any timepoint after correction.

### 3.5. Alpha Diversity

**Figure 5:**
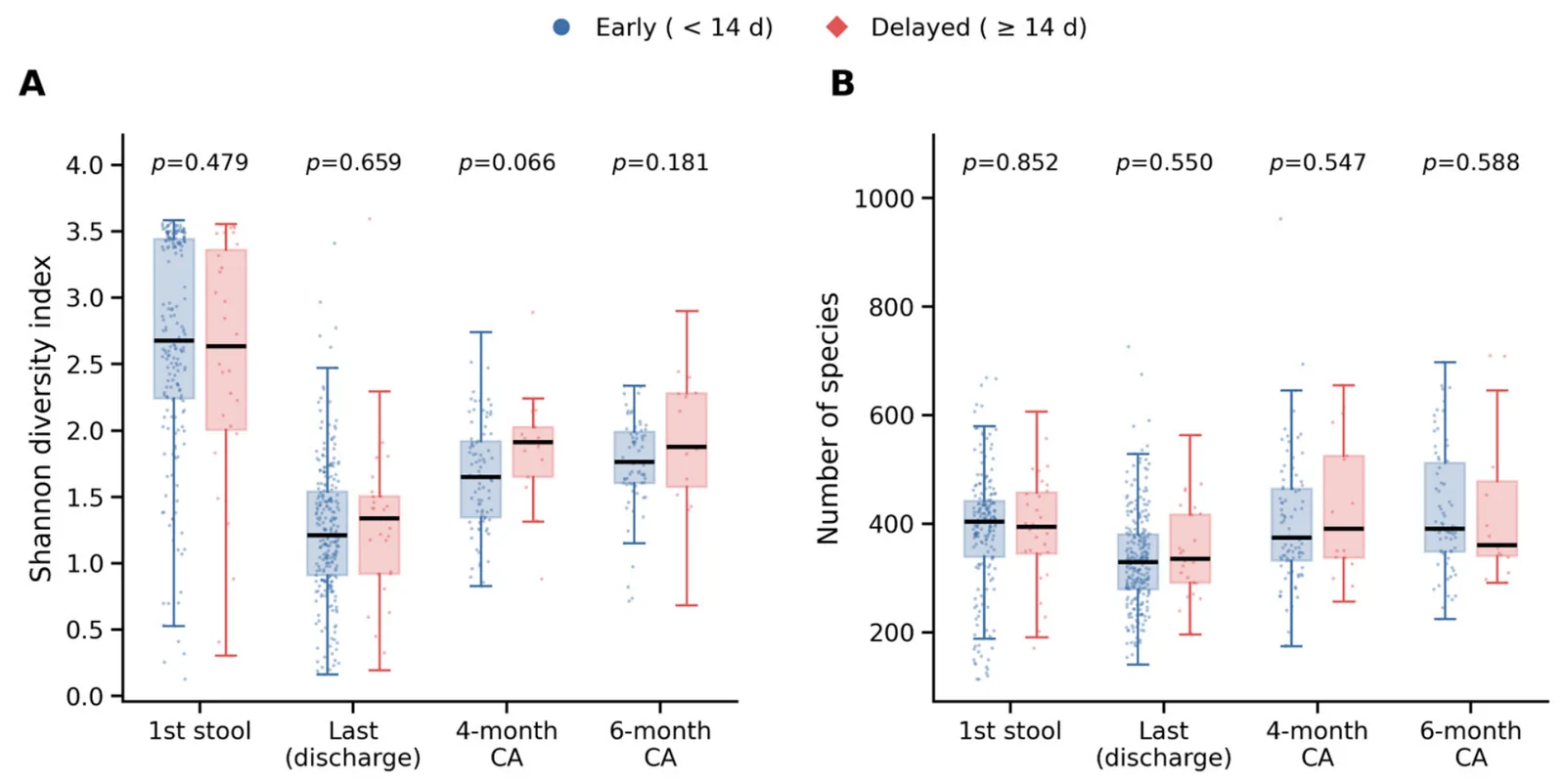
Alpha diversity of the gut microbiome in the Early FEF and Delayed FEF groups across four timepoints. (**A**) Shannon diversity index. (**B**) Number of species. Each box shows the median and interquartile range. The *p* value above each timepoint is the nominal *p* value from the Mann–Whitney U test comparing the two groups. Abbreviations: CA, corrected age; FEF, full enteral feeding.

Alpha diversity did not differ between the Early group and the Delayed group at any timepoint (all *p* > 0.05; Figure 5, Table S4). Median Shannon values for the Early group and the Delayed group were 2.68 [2.25–3.44] vs. 2.64 [2.01–3.36] at first stool (*p* = 0.479), 1.21 [0.91–1.54] vs. 1.34 [0.92–1.51] at discharge (*p* = 0.659), 1.65 [1.35–1.92] vs. 1.91 [1.65–2.03] at 4-month CA (*p* = 0.066), and 1.77 [1.60–1.99] vs. 1.88 [1.58–2.28] at 6-month CA (*p* = 0.181). Values were numerically higher in the Delayed group at 4-month CA and 6-month CA, but neither difference reached significance. The number of species identified also showed no between-group difference at any timepoint (all *p* > 0.05).

### 3.6. Beta Diversity

**Figure 6:**
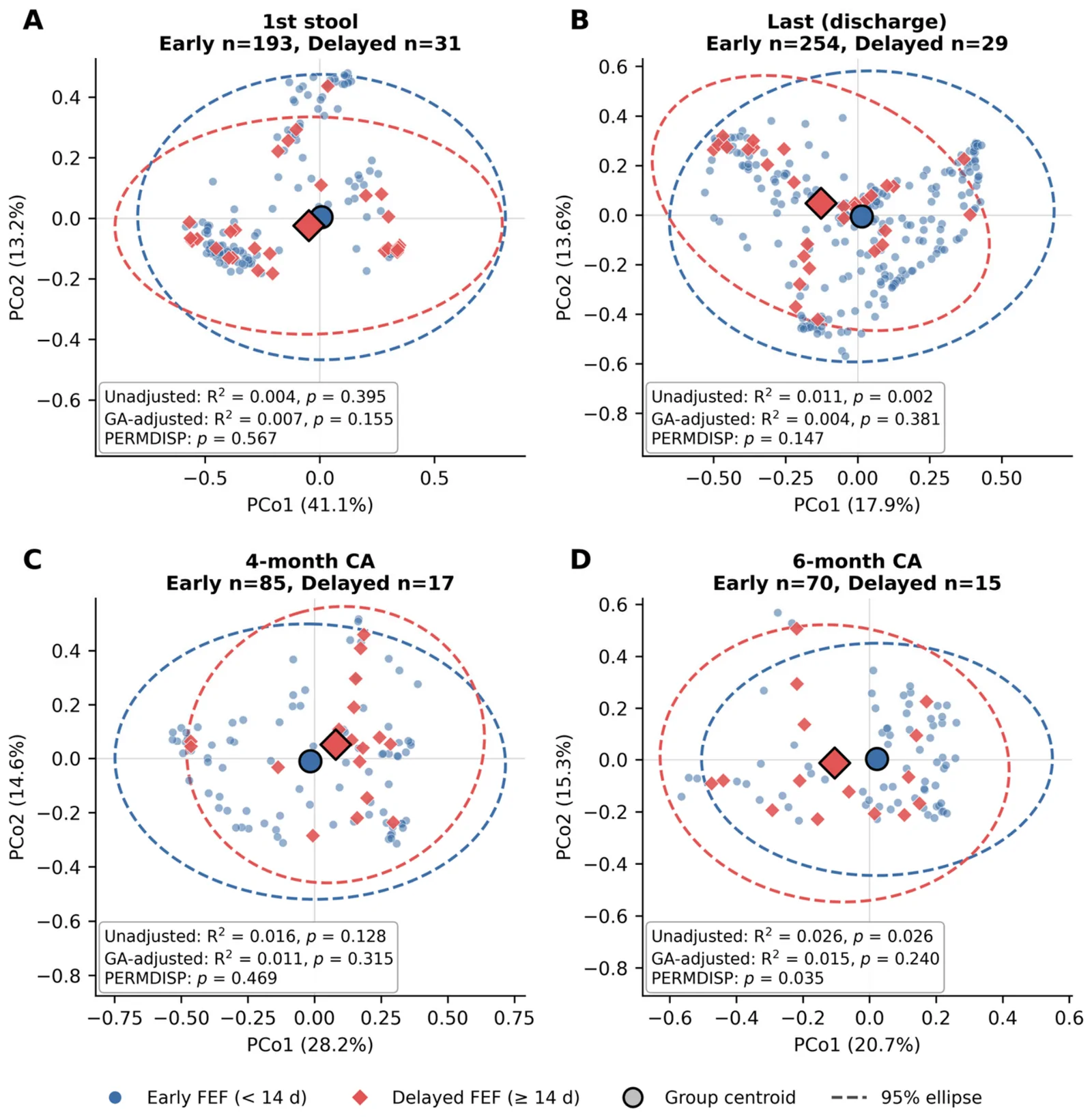
Beta diversity of the gut microbiome in the Early FEF and Delayed FEF groups, shown as principal coordinates analysis (PCoA) of genus-level Bray–Curtis dissimilarity. (**A**) First stool (Early FEF, n = 193; Delayed FEF, n = 31). (**B**) Last stool before NICU discharge (Early FEF, n = 254; Delayed FEF, n = 29). (**C**) 4-month CA (Early FEF, n = 85; Delayed FEF, n = 17). (**D**) 6-month CA (Early FEF, n = 70; Delayed FEF, n = 15); PERMDISP was significant at this timepoint, so the unadjusted PERMANOVA result may partly reflect unequal dispersion rather than a difference in group centroids. In each panel, each point represents one sample colored by FEF group, large symbols indicate group centroids, dashed lines indicate 95% ellipses, and the percentage of variance explained is shown on each axis. The box in each panel shows the unadjusted and GA-adjusted PERMANOVA results (9,999 permutations) and the PERMDISP *p* value for within-group dispersion. Abbreviations: CA, corrected age; FEF, full enteral feeding; GA, gestational age; NICU, neonatal intensive care unit; PCoA, principal coordinates analysis; PERMANOVA, permutational multivariate analysis of variance; PERMDISP, permutational analysis of multivariate dispersions.

Bray–Curtis dissimilarity was assessed by PCoA and PERMANOVA (9999 permutations; Figure 6, Table S4). At first stool, community composition did not differ between the Early group and the Delayed group (R^2^ = 0.004, *p* = 0.395). By discharge, the two groups differed in unadjusted analysis (R^2^ = 0.011, *p* = 0.002), with the first two PCoA axes explaining 17.9% and 13.6% of the variance. PERMDISP indicated comparable within-group dispersion at this timepoint (*p* = 0.147), so the PERMANOVA result reflects a difference in group centroids rather than in dispersion. The between-group difference was not significant at 4-month CA (R^2^ = 0.016, *p* = 0.128). At 6-month CA, a difference was again present in unadjusted analysis (R^2^ = 0.026, *p* = 0.026), but PERMDISP was also significant at this timepoint (*p* = 0.035), so this result may partly reflect unequal dispersion between groups. After adjustment for GA, community composition did not differ between groups at any timepoint (first stool *p* = 0.155, discharge *p* = 0.381, 4-month CA *p* = 0.315, and 6-month CA *p* = 0.240). The FEF group explained less than 3% of the variation in community composition at every timepoint (all R^2^ < 0.03).

### 3.7. Sensitivity Analysis at Discharge

At discharge, the lower *Streptococcus* abundance in the Delayed group was attenuated after adjustment for GA (β −0.94, 95% CI −1.97 to 0.09, *p* = 0.074; Table S5). In infants born before 34 weeks of gestation, all of whom received probiotics and antibiotics, the estimate remained in the same direction but was smaller (β −0.60, 95% CI −1.84 to 0.65). Additional adjustment for breast milk exposure did not change it materially (β −0.54, 95% CI −1.79 to 0.72). Bacteroidetes was higher in the Delayed group in all three models (β 0.67 to 0.77), and the confidence interval excluded zero only in the full cohort. After adjustment for GA, community composition at discharge did not differ between groups (PERMANOVA R^2^ = 0.004, *p* = 0.381), and the results were similar in the restricted cohort (*p* = 0.850 and *p* = 0.848).

## 4. Discussion

In this cohort of 328 NICU infants followed with stool sampling at four timepoints from first stool to 6-month corrected age, we examined whether the timing of FEF was associated with gut microbiome development. The two groups did not differ at first stool after correction for multiple testing, differed most at NICU discharge, and showed smaller differences thereafter. At the genus level, *Streptococcus* at discharge was the only taxon that differed between groups after correction for multiple comparisons, being lower in the Delayed group, although this difference was attenuated after adjustment for GA. At the phylum level, the higher Proteobacteria and lower Firmicutes seen in the Delayed group at discharge in unadjusted analysis were attenuated after adjustment for GA, and no phylum showed a GA-adjusted difference in its developmental trajectory. Alpha diversity was preserved at every timepoint, whereas beta diversity differed in unadjusted analysis at discharge and, more weakly, at 6-month corrected age, but not after adjustment for GA. When time to FEF was analyzed as a continuous variable in exploratory analyses, longer time to FEF was associated with higher Bacteroidetes abundance.

Two features distinguish this study from prior work. Most previous preterm cohorts were cross-sectional or confined to the NICU admission period, and few followed the same infants from birth into corrected infancy [14,15,16]. Our four-timepoint design captured both the admission period and the months after discharge, when feeding and environmental exposures change. In addition, gestational age and time to full enteral feeding are strongly correlated in NICU populations, yet earlier studies rarely separated the two. By adjusting for gestational age in the longitudinal and beta diversity analyses and by examining the feeding contrast explicitly, we could ask whether feeding timing carried information about microbiome development beyond gestational age itself. The findings were also examined in several sensitivity analyses, including a continuous exposure, restriction to infants born before 34 weeks of gestation, and GA-adjusted beta diversity.

In the unadjusted genus-level screen, the only difference that remained after correction was the lower abundance of *Streptococcus* in the Delayed group at discharge. *Streptococcus* belongs to the class Bacilli, which dominates the earliest stage of gut colonization in preterm infants [14]. One possible explanation, which we could not test, is that infants who reach full enteral feeding later spend longer on parenteral nutrition with minimal oral intake, which may delay the seeding of these organisms. Because this was a single-timepoint finding within a large set of comparisons, it should be regarded as a hypothesis-generating association rather than a definitive effect, and confirmation in independent cohorts is needed. In addition, the association was attenuated after adjustment for GA and was smaller in infants born before 34 weeks of gestation, so it could not be separated from maturity and from the exposures that accompany delayed feeding.

At the phylum level, the Delayed group showed higher Proteobacteria and lower Firmicutes at discharge in unadjusted comparisons, a pattern typical of the less mature preterm gut [6,7]. These differences did not persist after adjustment for gestational age, and no phylum showed a gestational age-adjusted trajectory difference across time. This result is consistent with the strong inverse correlation between gestational age and time to full enteral feeding in this cohort. Lower gestational age both delays enteral feeding and independently shapes early colonization, so the crude phylum differences cannot be attributed to feeding timing separately from maturity. Feeding timing may also lie on a pathway from gestational age to the microbiome, so that part of the association between gestational age and the microbiome operates through feeding timing [27]. We could not test this possibility directly. Formal mediation analysis would require a larger cohort with greater independent variation in feeding timing, and we regard it as a priority for future work rather than a conclusion of the present study. When time to FEF was analyzed as a continuous variable, longer time to FEF was associated with higher Bacteroidetes abundance at discharge and at 4-month corrected age. The estimate at first stool, which preceded enteral feeding, was in the same direction, so this association may partly reflect residual confounding by maturity rather than an effect of feeding timing. Given the strong correlation between GA and time to FEF and the absence of a significant FEF × time interaction, these findings should be considered exploratory.

We did not observe reduced *Bifidobacterium* colonization in the Delayed group, although delayed *Bifidobacterium* colonization has been associated with lower gestational age [6]. *Bifidobacterium* was higher in the Delayed group at discharge in the unadjusted screen (*p* = 0.030), although this difference did not survive correction, and it did not differ between groups at 4-month corrected age. This result does not support a broad feeding-related suppression of anaerobic commensals in our cohort, although the study had limited power to detect modest differences. The absence of this pattern may reflect the more frequent breast milk exposure in the Delayed group, because bifidobacteria are adapted to utilize human milk glycans [1].

Composition did not differ at first stool, but differed most at discharge in unadjusted analyses, and showed smaller differences after discharge. The discharge comparison should be interpreted with particular caution. At the discharge sample, infants in the Delayed group were older than those in the Early group (median postnatal age of 95 vs. 14 days, and postmenstrual age of 41.9 vs. 37.8 weeks). Adjustment for GA does not make the two sets of discharge samples developmentally equivalent, so differences at discharge may reflect postnatal maturation and longer exposure to the NICU environment as well as feeding timing. Alpha diversity was preserved throughout, so the differences that did appear reflected a shift in which taxa predominated rather than a loss of richness or evenness. Beta diversity supported this reading in unadjusted analysis, with a clear separation at discharge that was not explained by unequal within-group dispersion. This separation was no longer present after adjustment for GA. The weaker separation at 6-month corrected age in unadjusted analysis, which was not present after adjustment for GA, should be interpreted with caution, because within-group dispersion also differed at that timepoint, so the apparent difference may partly reflect heterogeneity rather than a shift in the community centroid. Taken together, between-group differences were most apparent during the NICU period and smaller after discharge. Post-discharge samples were fewer, and follow-up was more complete in more preterm infants, so the smaller differences after discharge should not be interpreted as recovery. Probiotic administration also stopped and feeding practices changed at discharge, so the smaller differences after discharge cannot be attributed to feeding timing alone.

These findings should be placed in clinical context with care. Because gestational age and time to full enteral feeding were closely linked, our data do not establish that advancing feeds independently improves the microbiome, and they do not support using microbiome endpoints to justify feeding decisions. Time to FEF is therefore better regarded as a clinical phenotype that reflects gestational maturity, feeding tolerance, illness severity, and the interventions that accompany them, rather than as an isolated exposure. Timely advancement of enteral feeding is already a clinical goal in preterm infants, and later achievement of full enteral feeding has been associated with a higher risk of late-onset sepsis [11], although faster advancement did not reduce late-onset sepsis in a large trial [10]. Our results add that, in unadjusted analyses, the period of delayed feeding coincided with the widest divergence in gut microbiome composition, including a lower abundance of *Streptococcus* at discharge, but that these differences were not independent of GA and were smaller after discharge. This temporal pattern identifies the NICU admission period as the window in which between-group microbiome differences are most apparent, and in which studies of feeding and probiotic strategies aimed at commensal colonization would be most informative [28]. Whether earlier feeding confers microbiome benefit beyond gestational age remains to be demonstrated, and until then these observations should not be used to modify existing feeding guidance.

Several NICU exposures accompanied delayed feeding and could contribute to the differences observed at discharge. Infants in the Delayed group received antibiotics for longer, and this exposure could not be separated from feeding timing in our data. Probiotic use was universal in infants born before 34 weeks of gestation, and restriction to this group did not reverse the direction of the discharge associations. The probiotic strain was *L. reuteri*, so a direct contribution of the administered organism to *Streptococcus* abundance is unlikely, although indirect ecological effects cannot be excluded. Breast milk exposure was more common in the Delayed group. *Streptococcus* is among the predominant genera in human milk [29], so confounding by breast milk would be expected to increase rather than decrease *Streptococcus* abundance in the Delayed group. The relationships between breast milk feeding, feeding advancement, and microbial colonization in preterm infants deserve study with detailed data on milk type and volume over time.

This study has several limitations. The 16S rRNA sequencing resolves taxa reliably only to the genus level and does not measure microbial function, so the substrate-based mechanisms outlined in the Introduction remain untested and will require metagenomic and metabolomic approaches. Sample sizes were smaller at the 4-month and 6-month timepoints, which reduced power after discharge and widened the confidence intervals for the later trajectories. Follow-up was more complete in more preterm infants and in the Delayed group, so the later timepoints over-represent these infants. The assumption that missingness depended only on variables in the model cannot be verified, and selection within groups cannot be excluded. The Delayed group was small (n = 35) and imbalanced relative to the Early group, and the 14-day threshold was partly data-driven, which limits the efficiency of between-group inference. Almost all infants in the Delayed group were born before 34 weeks of gestation, so GA-adjusted comparisons relied on limited overlap between groups and cannot exclude moderate independent effects of feeding timing. Information on sequencing depth and negative extraction controls was not available. Given the low-biomass nature of first-stool samples, contamination introduced during sample processing cannot be fully excluded. The absence of negative extraction controls limited our ability to distinguish true low-abundance microbial signals from potential reagent- or process-related contaminants. Therefore, findings involving low-abundance taxa should be interpreted cautiously. The strong collinearity between gestational age and feeding timing precludes full statistical separation of their effects, and the *Streptococcus* association, which was attenuated after adjustment for GA, rested on a single timepoint and should be confirmed. Species-level assignment from V3–V4 sequences is unreliable, so the number of species identified should be regarded as an approximate measure of richness. Antibiotic exposure was available only as cumulative days without administration dates, and data on feeding and probiotic use after discharge were not available. Residual confounding by antibiotic exposure, feeding type, probiotics, and illness severity is therefore possible. Finally, this was a single-center study, and feeding protocols, antibiotic stewardship, and probiotic policies vary across institutions. Future work should include formal mediation analysis with richer variation in feeding timing, multi-omics characterization of the proposed substrate pathways, and multicenter validation.

## 5. Conclusions

In this cohort of NICU infants followed to 6-month corrected age, the microbiome differences observed with delayed full enteral feeding, including lower *Streptococcus* abundance and a different community structure at discharge, were not independent of gestational age. In exploratory analyses, longer time to full enteral feeding was associated with higher Bacteroidetes abundance, although residual confounding cannot be excluded. Time to full enteral feeding appears to reflect a clinical phenotype of maturity and illness severity rather than an isolated exposure, and whether earlier feeding benefits the microbiome independently of maturity remains to be determined.

## Figures and Tables

**Table 1:** Demographic and clinical characteristics of the study population.

Characteristic	Early FEF (n = 293)	Delayed FEF (n = 35)	*p*-Value
GA (weeks)	35.3 (33.9–37.4)	27.9 (26.2–30.0)	<0.001
Birth weight (g)	2370 (2011–2910)	1005 (762.5–1339)	<0.001
Male sex	147 (50.2)	15 (42.9)	0.413
Cesarean section	268 (91.8)	34 (97.1)	0.259
ACS	158 (53.9)	34 (97.1)	<0.001
PPROM	34 (11.6)	10 (28.6)	0.005
HCAM	6 (2.0)	6 (17.1)	<0.001
SGA	28 (9.6)	9 (25.7)	0.004
RDS	75 (25.6)	29 (82.9)	<0.001
Symptomatic PDA	0 (0.0)	15 (42.9)	<0.001
BPD	3 (1.0)	13 (37.1)	<0.001
NEC or FI	23 (7.8)	10 (28.6)	<0.001
Culture-proven sepsis	3 (1.0)	9 (25.7)	<0.001
IVH grade ≥ 2	2 (0.7)	15 (42.9)	<0.001
ROP requiring treatment	2 (0.7)	8 (22.9)	<0.001
PVL	66 (22.5)	28 (80.0)	<0.001
Length of hospital stay (days)	15 (10–25)	109 (68.5–138.5)	<0.001
Duration of mechanical ventilation (days)	0 (0–2)	11 (2–30.5)	<0.001
TPN duration (days)	3 (2–5)	36 (25.5–56.5)	<0.001
Day to FEF (days)	5 (4–6)	27 (18.5–42)	<0.001
Probiotics use	194 (66.2)	35 (100.0)	<0.001
Antibiotic use	287 (98.0)	35 (100.0)	1.000
Antibiotic days	8 (7–11)	45 (30.5–95.5)	<0.001
Breast milk > 50%	7 (2.4)	13 (37.1)	<0.001

Data are presented as median (interquartile range) for continuous variables or n (%) for categorical variables. *p*-values were obtained using the Mann–Whitney U test for continuous variables and the chi-square or Fisher exact test for categorical variables. Bold indicates *p* < 0.05. Antibiotic days is the sum of the days of administration of each antibiotic agent during admission. Breast milk > 50% indicates that breast milk accounted for more than 50% of the total enteral intake during admission. Abbreviations: ACS, antenatal corticosteroid; BPD, bronchopulmonary dysplasia; FEF, full enteral feeding; FI, feeding intolerance; GA, gestational age; HCAM, histological chorioamnionitis; IVH, intraventricular hemorrhage; NEC, necrotizing enterocolitis; PDA, patent ductus arteriosus; PPROM, preterm premature rupture of membranes; PVL, periventricular leukomalacia; RDS, respiratory distress syndrome; ROP, retinopathy of prematurity; SGA, small for gestational age; TPN, total parenteral nutrition.

## Data Availability

The datasets analyzed in this study are not publicly available. The information contained in the data must be protected as confidential and will only become available to those individuals who have obtained permission from the data review board and IRB of Seoul St. Mary’s Hospital to access and use the data for permitted research activities. The original contributions presented in this study are included in the article materials. Further inquiries can be directed to the corresponding authors.

## Supplementary Materials

The following supporting information can be downloaded at: https://www.mdpi.com/article/10.3390/nu18193262/s1; Table S1: Phylum-level relative abundance (%) by FEF group at each sampling timepoint; Table S2: Genus-level relative abundance (%) by full enteral feeding (FEF) group at each sampling timepoint; Table S3: Longitudinal phylum-level gut microbiome composition by timing of full enteral feeding, estimated with linear mixed models adjusted for gestational age and gestational age × timepoint.; Table S4: Alpha and beta diversity of the gut microbiome by full enteral feeding (FEF) group at each sampling timepoint; Table S5: Sensitivity analysis of gut microbiome composition at NICU discharge in the Delayed FEF group compared with the Early FEF group, with restriction to infants born before 34 weeks of gestation and adjustment for breast milk exposure.

## References

[1] Milani C., Duranti S., Bottacini F., Casey E., Turroni F., Mahony J., Belzer C., Delgado Palacio S., Arboleya Montes S., Mancabelli L. (2017). The First Microbial Colonizers of the Human Gut: Composition, Activities, and Health Implications of the Infant Gut Microbiota. Microbiol. Mol. Biol. Rev..

[2] Tamburini S., Shen N., Wu H.C., Clemente J.C. (2016). The microbiome in early life: Implications for health outcomes. Nat. Med..

[3] Shao Y., Forster S.C., Tsaliki E., Vervier K., Strang A., Simpson N., Kumar N., Stares M.D., Rodger A., Brocklehurst P. (2019). Stunted microbiota and opportunistic pathogen colonization in caesarean-section birth. Nature.

[4] Gibson M.K., Wang B., Ahmadi S., Burnham C.A., Tarr P.I., Warner B.B., Dantas G. (2016). Developmental dynamics of the preterm infant gut microbiota and antibiotic resistome. Nat. Microbiol..

[5] Henderickx J.G.E., Zwittink R.D., van Lingen R.A., Knol J., Belzer C. (2019). The Preterm Gut Microbiota: An Inconspicuous Challenge in Nutritional Neonatal Care. Front. Cell. Infect. Microbiol..

[6] Korpela K., Blakstad E.W., Moltu S.J., Strømmen K., Nakstad B., Rønnestad A.E., Brække K., Iversen P.O., Drevon C.A., de Vos W. (2018). Intestinal microbiota development and gestational age in preterm neonates. Sci. Rep..

[7] Underwood M.A., Sohn K. (2017). The Microbiota of the Extremely Preterm Infant. Clin. Perinatol..

[8] Pammi M., Cope J., Tarr P.I., Warner B.B., Morrow A.L., Mai V., Gregory K.E., Kroll J.S., McMurtry V., Ferris M.J. (2017). Intestinal dysbiosis in preterm infants preceding necrotizing enterocolitis: A systematic review and meta-analysis. Microbiome.

[9] Warner B.B., Deych E., Zhou Y., Hall-Moore C., Weinstock G.M., Sodergren E., Shaikh N., Hoffmann J.A., Linneman L.A., Hamvas A. (2016). Gut bacteria dysbiosis and necrotising enterocolitis in very low birthweight infants: A prospective case-control study. Lancet.

[10] Dorling J., Abbott J., Berrington J., Bosiak B., Bowler U., Boyle E., Embleton N., Hewer O., Johnson S., Juszczak E. (2019). Controlled Trial of Two Incremental Milk-Feeding Rates in Preterm Infants. N. Engl. J. Med..

[11] Salas A.A., Wiener L.E., Trotta M., Valcarce V., Romero-Lopez M., Ortigoza E.B., Fu T.T., McNelis K., Poindexter B., Carlo W.A. (2025). Time to Full Enteral Feeds and Late-Onset Sepsis in Extremely Preterm Infants. JAMA Netw. Open.

[12] Burrin D.G., Stoll B. (2002). Key nutrients and growth factors for the neonatal gastrointestinal tract. Clin. Perinatol..

[13] Grier A., Qiu X., Bandyopadhyay S., Holden-Wiltse J., Kessler H.A., Gill A.L., Hamilton B., Huyck H., Misra S., Mariani T.J. (2017). Impact of prematurity and nutrition on the developing gut microbiome and preterm infant growth. Microbiome.

[14] La Rosa P.S., Warner B.B., Zhou Y., Weinstock G.M., Sodergren E., Hall-Moore C.M., Stevens H.J., Bennett W.E., Shaikh N., Linneman L.A. (2014). Patterned progression of bacterial populations in the premature infant gut. Proc. Natl. Acad. Sci. USA.

[15] Stewart C.J., Embleton N.D., Marrs E.C.L., Smith D.P., Fofanova T., Nelson A., Skeath T., Perry J.D., Petrosino J.F., Berrington J.E. (2017). Longitudinal development of the gut microbiome and metabolome in preterm neonates with late onset sepsis and healthy controls. Microbiome.

[16] Ho T.T.B., Groer M.W., Kane B., Yee A.L., Torres B.A., Gilbert J.A., Maheshwari A. (2018). Dichotomous development of the gut microbiome in preterm infants. Microbiome.

[17] Shin J., Choi C.W., Kang H.M., Kim S.Y., Youn Y.A. (2025). Early-Life Development of the Intestinal Microbiome in Preterm and Term Infants Hospitalized in the Neonatal Intensive Care Unit. Microorganisms.

[18] Yoon B.H., Romero R., Kim C.J., Jun J.K., Gomez R., Choi J.H., Syn H.C. (1995). Amniotic fluid interleukin-6: A sensitive test for antenatal diagnosis of acute inflammatory lesions of preterm placenta and prediction of perinatal morbidity. Am. J. Obstet. Gynecol..

[19] Fenton T.R., Kim J.H. (2013). A systematic review and meta-analysis to revise the Fenton growth chart for preterm infants. BMC Pediatr..

[20] Jobe A.H., Bancalari E. (2001). Bronchopulmonary dysplasia. Am. J. Respir. Crit. Care Med..

[21] Walsh M.C., Kliegman R.M. (1986). Necrotizing enterocolitis: Treatment based on staging criteria. Pediatr. Clin. N. Am..

[22] Carlos M.A., Babyn P.S., Marcon M.A., Moore A.M. (1997). Changes in gastric emptying in early postnatal life. J. Pediatr..

[23] The Committee for the Classification of Retinopathy of Prematurity (1984). An international classification of retinopathy of prematurity. Arch. Ophthalmol..

[24] International Committee for the Classification of Retinopathy of Prematurity (2005). The international classification of retinopathy of prematurity revisited. Arch. Ophthalmol..

[25] Gloor G.B., Macklaim J.M., Pawlowsky-Glahn V., Egozcue J.J. (2017). Microbiome Datasets Are Compositional: And This Is Not Optional. Front. Microbiol..

[26] Schisterman E.F., Cole S.R., Platt R.W. (2009). Overadjustment bias and unnecessary adjustment in epidemiologic studies. Epidemiology.

[27] VanderWeele T.J. (2016). Mediation Analysis: A Practitioner’s Guide. Annu. Rev. Public Health.

[28] Morgan R.L., Preidis G.A., Kashyap P.C., Weizman A.V., Sadeghirad B. (2020). Probiotics Reduce Mortality and Morbidity in Preterm, Low-Birth-Weight Infants: A Systematic Review and Network Meta—Analysis of Randomized Trials. Gastroenterology.

[29] Fitzstevens J.L., Smith K.C., Hagadorn J.I., Caimano M.J., Matson A.P., Brownell E.A. (2017). Systematic Review of the Human Milk Microbiota. Nutr. Clin. Pract..

