# Supplementary material for: Timing of Full Enteral Feeding and Longitudinal Gut Microbiome Development in Infants Admitted to the Neonatal Intensive Care Unit
Source: Nutrients. 2026 Oct 2;18(19):3262. doi: 10.3390/nu18193262 (PMC13666783; doi:10.3390/nu18193262)
Supplement: Supplementary file 1 [file nutrients-18-03262-s001.zip › nutrients-4580025-supplementary.pdf]

Table S1. Phylum-level relative abundance (%) by FEF group at each sampling timepoint.

**1st stool**	**Early FEF (n=193)**	**Delayed FEF (n=31)**	***p*-value**
Firmicutes	34.4 ± 20.5	34.6 ± 23.3	0.786
Proteobacteria	41.4 ± 30.9	38.6 ± 32.8	0.470
Actinobacteria	5.7 ± 7.2	4.7 ± 3.9	0.457
Bacteroidetes	15.9 ± 12.1	15.0 ± 12.4	0.795
Verrucomicrobia	1.1 ± 2.5	0.8 ± 0.8	0.554
**Discharge**	**Early FEF (n=254)**	**Delayed FEF (n=29)**	***p*-value**
Firmicutes	50.2 ± 36.2	32.0 ± 31.0	0.012
Proteobacteria	43.8 ± 37.9	59.5 ± 34.3	0.035
Actinobacteria	3.9 ± 9.0	7.1 ± 16.9	0.581
Bacteroidetes	1.9 ± 5.8	1.2 ± 5.1	0.892
Verrucomicrobia	0.1 ± 0.2	0.1 ± 0.3	0.621
**4-month CA**	**Early FEF (n=85)**	**Delayed FEF (n=17)**	***p*-value**
Firmicutes	35.3 ± 20.4	39.1 ± 17.7	0.389
Proteobacteria	33.0 ± 20.1	30.2 ± 21.0	0.584
Actinobacteria	23.8 ± 25.2	13.6 ± 14.8	0.269
Bacteroidetes	1.8 ± 7.4	7.0 ± 14.9	0.578
Verrucomicrobia	5.9 ± 17.3	10.0 ± 20.5	0.615
**6-month CA**	**Early FEF (n=70)**	**Delayed FEF (n=15)**	***p*-value**
Firmicutes	55.3 ± 19.9	50.2 ± 20.4	0.319
Proteobacteria	31.3 ± 16.6	26.0 ± 19.3	0.319
Actinobacteria	9.9 ± 11.9	10.4 ± 13.5	0.493
Bacteroidetes	0.9 ± 4.6	5.0 ± 13.2	0.691
Verrucomicrobia	2.4 ± 9.9	8.3 ± 18.7	0.251

Data are presented as mean ± standard deviation. *p*-values are from the Mann-Whitney U test comparing the Early FEF and Delayed FEF groups at each timepoint. The number of samples contributing to each timepoint is shown. Means are presented because the medians of less abundant phyla were close to zero. p-values are nominal and were not corrected for multiple testing.

Abbreviations: CA, corrected age; FEF, full enteral feeding.

Table S2. Genus-level relative abundance (%) by full enteral feeding (FEF) group at each sampling timepoint.

Genus	Early group	Delayed group	*p*	*q*
**1st stool**	**n = 193**	**n = 31**		
Streptococcus	13.0 ± 12.1	11.2 ± 9.6	0.356	0.811
Veillonella	4.1 ± 6.3	2.6 ± 3.3	0.258	0.811
Enterococcus	1.8 ± 8.4	4.4 ± 10.6	0.691	0.821
Bifidobacterium	4.6 ± 4.1	3.8 ± 3.9	0.323	0.811
Escherichia/Shigella	2.0 ± 8.2	0.8 ± 0.7	0.106	0.631
Enterobacter	0.7 ± 3.3	0.2 ± 0.2	0.013	0.280
Klebsiella	0.3 ± 1.0	1.4 ± 6.9	0.139	0.706
Ralstonia	11.7 ± 17.4	13.0 ± 17.3	0.600	0.811
Bacteroides	8.7 ± 7.5	8.4 ± 7.8	0.809	0.891
Staphylococcus	3.3 ± 9.0	6.8 ± 17.9	0.603	0.811
**Discharge**	**n = 254**	**n = 29**		
Streptococcus	19.6 ± 26.1	6.2 ± 10.7	<0.001	0.028
Veillonella	3.5 ± 11.9	7.5 ± 17.4	0.340	0.811
Enterococcus	18.0 ± 26.8	7.1 ± 11.4	0.488	0.811
Bifidobacterium	2.3 ± 7.3	7.0 ± 17.4	0.030	0.379
Escherichia/Shigella	5.8 ± 18.7	11.3 ± 25.5	0.080	0.606
Enterobacter	12.6 ± 23.8	19.1 ± 28.4	0.139	0.706
Klebsiella	12.4 ± 26.3	10.9 ± 23.8	0.619	0.811
Ralstonia	0.9 ± 4.2	0.9 ± 3.6	0.586	0.811
Bacteroides	1.3 ± 5.1	0.7 ± 2.9	0.872	0.920
Staphylococcus	4.4 ± 14.3	2.0 ± 9.4	0.017	0.280
**4-month CA**	**n = 85**	**n = 17**		
Streptococcus	0.9 ± 2.2	0.6 ± 1.4	0.384	0.811
Veillonella	21.2 ± 20.3	20.7 ± 12.5	0.686	0.821
Enterococcus	2.6 ± 4.2	1.6 ± 2.6	0.414	0.811
Bifidobacterium	23.3 ± 24.3	13.4 ± 14.5	0.269	0.811
Escherichia/Shigella	13.3 ± 16.5	11.1 ± 19.3	0.641	0.811
Enterobacter	4.0 ± 8.1	3.7 ± 6.4	0.489	0.811
Klebsiella	4.2 ± 7.5	4.7 ± 9.8	0.893	0.930
Ralstonia	1.2 ± 5.4	0.1 ± 0.2	0.175	0.782
Bacteroides	1.4 ± 6.7	4.2 ± 12.0	0.822	0.893
Staphylococcus	0.4 ± 2.4	0.0 ± 0.0	0.559	0.811
**6-month CA**	**n = 70**	**n = 15**		
Streptococcus	0.7 ± 1.7	0.5 ± 1.4	0.242	0.811
Veillonella	36.6 ± 19.4	23.8 ± 15.8	0.018	0.280

Genus	Early group	Delayed group	*p*	*q*
Enterococcus	1.2 ± 3.9	3.4 ± 8.9	0.324	0.811
Bifidobacterium	9.9 ± 12.1	10.0 ± 12.8	0.471	0.811
Escherichia/Shigella	14.4 ± 17.3	11.0 ± 15.8	0.330	0.811
Enterobacter	2.5 ± 6.0	1.3 ± 2.0	0.568	0.811
Klebsiella	2.3 ± 5.3	6.9 ± 11.4	0.372	0.811
Ralstonia	0.2 ± 0.9	0.2 ± 0.4	0.590	0.811
Bacteroides	0.3 ± 1.4	4.9 ± 13.4	0.403	0.811
Staphylococcus	0.0 ± 0.0	0.0 ± 0.0	0.515	0.811

CA, corrected age; FEF, full enteral feeding; FDR, false discovery rate.
Data are presented as mean ± standard deviation for the ten genera with the highest mean relative abundance across the cohort. Genus-level medians were predominantly zero, so the mean is reported as the summary statistic.
The *p* values are from the Mann-Whitney U test comparing the Early group and the Delayed group at each timepoint. The *q* values are Benjamini-Hochberg FDR adjusted *p* values across the full testing family of 76 tests, comprising the 19 genera with a mean relative abundance of at least 1% across the cohort at each of the four timepoints. The nine genera not shown here are included in that correction but are omitted from the table for brevity. Significance was set at $q < 0.05$.
Bold type indicates the single comparison that remained significant after FDR correction.

Table S3. Longitudinal phylum-level gut microbiome composition by timing of full enteral feeding, estimated with linear mixed models adjusted for gestational age and gestational age × timepoint.

Phylum	Timepoint (n, Early/Delayed)	Delayed vs. Early FEF (primary)			Time to FEF, per doubling of days		
		β (95% CI)	*p*	*q*	β (95% CI)	*p*	*q*
Firmicutes	1st stool (193/31)	−0.06 (−0.67, 0.55)	0.854	0.943	−0.06 (−0.29, 0.17)	0.605	0.756
	Last (discharge) (254/29)	−0.25 (−0.82, 0.31)	0.375	0.689	−0.12 (−0.31, 0.08)	0.235	0.606
	4-month CA (85/17)	−0.30 (−1.12, 0.52)	0.470	0.723	−0.08 (−0.47, 0.31)	0.677	0.770
	6-month CA (70/15)	−0.58 (−1.48, 0.33)	0.211	0.689	−0.14 (−0.55, 0.26)	0.486	0.725
	FEF × time, χ^2 (3 df)	0.95	0.813	0.813	0.20	0.978	0.978
Proteobacteria	1st stool (193/31)	−0.65 (−1.60, 0.30)	0.180	0.689	−0.22 (−0.58, 0.15)	0.242	0.606
	Last (discharge) (254/29)	−0.39 (−1.26, 0.48)	0.379	0.689	−0.14 (−0.44, 0.17)	0.378	0.712
	4-month CA (85/17)	0.05 (−1.20, 1.29)	0.943	0.943	−0.11 (−0.70, 0.47)	0.708	0.770
	6-month CA (70/15)	−0.80 (−2.17, 0.57)	0.251	0.689	−0.34 (−0.95, 0.27)	0.280	0.621
	FEF × time, χ^2 (3 df)	1.29	0.731	0.813	0.50	0.919	0.978
Actinobacteria	1st stool (193/31)	0.16 (−0.52, 0.84)	0.642	0.917	0.10 (−0.15, 0.36)	0.427	0.712
	Last (discharge) (254/29)	−0.06 (−0.68, 0.56)	0.858	0.943	−0.14 (−0.36, 0.07)	0.187	0.606
	4-month CA (85/17)	0.41 (−0.49, 1.32)	0.370	0.689	−0.17 (−0.60, 0.26)	0.427	0.712
	6-month CA (70/15)	−0.40 (−1.40, 0.60)	0.433	0.722	0.08 (−0.37, 0.53)	0.733	0.770
	FEF × time, χ^2 (3 df)	1.72	0.632	0.813	2.92	0.404	0.862
Bacteroidetes	1st stool (193/31)	0.43 (−0.20, 1.05)	0.180	0.689	0.20 (−0.03, 0.44)	0.093	0.465
	Last (discharge) (254/29)	0.72 (0.15, 1.29)	**0.014**	0.140	0.28 (0.09, 0.48)	**0.005**	**0.045**
	4-month CA (85/17)	0.69 (−0.14, 1.52)	0.102	0.681	0.74 (0.35, 1.13)	**<0.001**	**0.004**
	6-month CA (70/15)	−0.05 (−0.96, 0.87)	0.919	0.943	0.54 (0.13, 0.94)	**0.010**	0.065
	FEF × time, χ^2 (3 df)	2.37	0.498	0.813	6.83	0.077	0.387
Verrucomicrobia	1st stool (193/31)	0.19 (−0.82, 1.21)	0.709	0.943	−0.06 (−0.45, 0.33)	0.770	0.770
	Last (discharge) (254/29)	−0.05 (−0.98, 0.89)	0.920	0.943	0.11 (−0.22, 0.43)	0.523	0.725
	4-month CA (85/17)	−0.69 (−2.05, 0.67)	0.323	0.689	−0.41 (−1.05, 0.24)	0.221	0.606
	6-month CA (70/15)	1.98 (0.48, 3.48)	**0.010**	0.140	−0.21 (−0.89, 0.47)	0.544	0.725
	FEF × time, χ^2 (3 df)	7.70	0.053	0.264	2.28	0.517	0.862

CLR was computed across the five major phyla with a pseudocount of 0.0001. Each phylum was analyzed with a linear mixed model including the FEF exposure, timepoint, FEF exposure × timepoint, GA, and GA × timepoint as fixed effects and a random intercept for each infant, fitted by restricted maximum likelihood. The GA × timepoint terms allowed the association between GA and each phylum to differ by timepoint. In the primary analysis, β is the difference in CLR-transformed relative abundance between the Delayed FEF group and the Early FEF group (Delayed minus Early). In the sensitivity analysis, time to FEF was entered as log2(days), and β is the difference in CLR-transformed relative abundance per doubling of days to FEF. Timepoint-specific estimates were tested with Wald z tests. The FEF × time row is the joint Wald test of the three interaction terms. q values are Benjamini-Hochberg adjusted within each analysis, across the 20 timepoint-specific estimates and separately across the five interaction tests. Bold indicates p or $q < 0.05$. In the model used in the original submission, which included GA as a single term, no FEF × time interaction remained significant after correction (minimum $q = 0.195$). Seven of 20 timepoint-specific contrasts reached nominal significance, including contrasts at first stool (Proteobacteria, $p = 0.039$; Bacteroidetes, $p = 0.021$), and none remained significant after correction (minimum $q = 0.078$). The analysis included 328 infants (Early FEF, n=293; Delayed FEF, n=35) and 694 stool samples. Median time to FEF was 5 days (IQR 4–6; range 1–134). The Spearman correlation between GA and time to FEF was $\rho = -0.62$ ($p < 0.001$).

Abbreviations: CA, corrected age; CI, confidence interval; CLR, centered log-ratio; FEF, full enteral feeding; GA, gestational age; IQR, interquartile range.

Table S4. Alpha and beta diversity of the gut microbiome by full enteral feeding (FEF) group at each sampling timepoint.

(A) Alpha diversity

Index	Timepoint (n, Early/Delayed)	Early FEF	Delayed FEF	*p*
Shannon index	1st stool (193/31)	2.68 (2.25–3.44)	2.64 (2.01–3.36)	0.479
	Last (discharge) (254/29)	1.21 (0.91–1.54)	1.34 (0.92–1.51)	0.659
	4-month CA (85/17)	1.65 (1.35–1.92)	1.91 (1.65–2.03)	0.066
	6-month CA (70/15)	1.77 (1.60–1.99)	1.88 (1.58–2.28)	0.181
Number of species identified	1st stool (193/31)	405 (340–443)	395 (346–458)	0.852
	Last (discharge) (254/29)	330 (280–382)	336 (292–418)	0.550
	4-month CA (85/17)	375 (333–465)	392 (338–526)	0.547
	6-month CA (70/15)	392 (350–513)	361 (341–479)	0.588

(B) Beta diversity

Timepoint	Samples, Early/Delayed	PERMANOVA, unadjusted		PERMANOVA, GA-adjusted		PERMDISP
		R^2	*p*	R^2	*p*	*p*
1st stool	193 / 31	0.004	0.395	0.007	0.155	0.567
Last (discharge)	254 / 29	0.011	**0.002**	0.004	0.381	0.147
4-month CA	85 / 17	0.016	0.128	0.011	0.315	0.469
6-month CA	70 / 15	0.026	**0.026**	0.015	0.240	**0.035**

Alpha diversity data are presented as median (interquartile range), and p values are from the Mann-Whitney U test. The number of species identified is based on species-level assignment of V3–V4 sequences and is an approximate measure of richness. Beta diversity was assessed with genus-level Bray-Curtis dissimilarity. PERMANOVA was performed without adjustment and with GA as a covariate, testing the FEF group term conditional on GA by Freedman-Lane permutation of residuals from the GA-only model. R^2 is the proportion of total variation attributable to FEF group. PERMDISP compared distances to the group centroid in principal coordinate space. All permutation tests used 9,999 permutations. At 6-month CA the PERMDISP result was significant, so the unadjusted PERMANOVA result at that timepoint may partly reflect unequal dispersion rather than a difference in group centroids. Bold indicates $p < 0.05$.

Abbreviations: CA, corrected age; FEF, full enteral feeding; GA, gestational age; PERMANOVA, permutational multivariate analysis of variance; PERMDISP, permutational analysis of multivariate dispersions.

Table S5. Sensitivity analysis of gut microbiome composition at NICU discharge in the Delayed FEF group compared with the Early FEF group, with restriction to infants born before 34 weeks of gestation and adjustment for breast milk exposure.

**Outcome**	**Full cohort, adjusted for GA (254/29)**		**GA < 34 weeks, adjusted for GA (70/29)**		**GA < 34 weeks, adjusted for GA and breast milk (70/29)**	
	**β (95% CI)**	***p***	**β (95% CI)**	***p***	**β (95% CI)**	***p***
Genus, CLR						
Streptococcus	−0.94 (−1.97, 0.09)	0.074	−0.60 (−1.84, 0.65)	0.342	−0.54 (−1.79, 0.72)	0.399
Phylum, CLR						
Firmicutes	−0.26 (−0.96, 0.45)	0.473	0.08 (−0.84, 1.00)	0.861	0.10 (−0.87, 1.07)	0.835
Proteobacteria	−0.37 (−1.36, 0.62)	0.461	−0.40 (−1.52, 0.72)	0.482	−0.40 (−1.54, 0.74)	0.488
Actinobacteria	−0.05 (−0.86, 0.76)	0.906	−0.30 (−1.26, 0.66)	0.541	−0.41 (−1.54, 0.73)	0.479
Bacteroidetes	0.77 (0.22, 1.32)	**0.007**	0.67 (−0.04, 1.37)	0.063	0.71 (−0.05, 1.47)	0.066
Verrucomicrobia	−0.09 (−0.97, 0.79)	0.842	−0.05 (−1.28, 1.17)	0.932	−0.01 (−1.33, 1.32)	0.993
Beta diversity, Bray-Curtis						
PERMANOVA, R^2	0.0036	0.381	0.0055	0.850	0.0055	0.848
PERMDISP, F	2.08	0.147	4.12	0.050	4.12	0.050

Analyses were restricted to the last stool sample before NICU discharge. Numbers in the column headers are samples in the Early FEF and Delayed FEF groups. β is the difference in CLR-transformed relative abundance between the Delayed FEF group and the Early FEF group (Delayed minus Early), estimated by linear regression with heteroscedasticity-consistent (HC3) standard errors. For *Streptococcus*, CLR was computed across the 19 genera with a cohort mean relative abundance of at least 1% and the sum of all remaining genera. For phyla, CLR was computed across the five major phyla. A pseudocount of 0.0001 was used. Breast milk was entered as receipt of breast milk for more than 50% of the total enteral intake during admission. In infants born before 34 weeks of gestation, all infants received probiotics and antibiotics during admission. Median GA was 33.1 weeks in the Early FEF group and 28.7 weeks in the Delayed FEF group. Breast milk for more than 50% of intake was recorded in 5 and 10 infants, respectively. PERMANOVA tested the FEF group term conditional on the covariates, using Freedman-Lane permutation of residuals from the covariate-only model (9,999 permutations). R^2 is the proportion of total variation attributable to FEF group. PERMDISP compared distances to the group centroid in principal coordinate space (9,999 permutations) and was not covariate adjusted. p values are nominal and were not corrected for multiple testing. Bold indicates $p < 0.05$.

Abbreviations: CI, confidence interval; CLR, centered log-ratio; FEF, full enteral feeding; GA, gestational age; NICU, neonatal intensive care unit; PERMANOVA, permutational multivariate analysis of variance; PERMDISP, permutational analysis of multivariate dispersions.
